# Protein Digestion-Derived Peptides and the Peripheral Regulation of Food Intake

**DOI:** 10.3389/fendo.2017.00085

**Published:** 2017-04-24

**Authors:** Juliette Caron, Dorothée Domenger, Pascal Dhulster, Rozenn Ravallec, Benoit Cudennec

**Affiliations:** ^1^Université Lille, INRA, Université Artois, Université Littoral Côte d’Opale, EA 7394 – ICV – Institut Charles Viollette, Lille, France

**Keywords:** protein digestion, bioactive peptides, food intake regulation, gut hormones, dipeptidyl peptidase IV, enteroendocrine cells

## Abstract

The gut plays a central role in energy homeostasis. Food intake regulation strongly relies on the gut–brain axis, and numerous studies have pointed out the significant role played by gut hormones released from enteroendocrine cells. It is well known that digestive products of dietary protein possess a high satiating effect compared to carbohydrates and fat. Nevertheless, the processes occurring in the gut during protein digestion involved in the short-term regulation of food intake are still not totally unraveled. This review provides a concise overview of the current data concerning the implication of food-derived peptides in the peripheral regulation of food intake with a focus on the gut hormones cholecystokinin and glucagon-like peptide 1 regulation and the relationship with some aspects of glucose homeostasis.

## Introduction

Food intake regulation strongly relies on the gut–brain axis, and numerous studies have pointed out the significant role played by gut hormones in response to food digestion (1, 2). These hormones are involved in appetite regulation as short-term peripheral satiety signals. They promote satiety, i.e., diminish appetite and reduce food intake by endocrine and nervous paths activating different signaling pathways (3–5).

The increasing expansion of obesity-related diseases has led the scientific community to explore new therapeutic approaches. They need to promote long-term body weight decrease and stabilization, especially fat loss, as well as satiety while reducing caloric intake (6). Dietary proteins have a greater satiety effect than carbohydrates and fat when equally consumed (7). However, this effect may rely on the protein source (8). Satiating properties of dietary proteins come from various physiological effects such as gut hormone secretion stimulation, energy expenditure and amino acid circulating level increase, and gluconeogenesis stimulation (9). Nevertheless, the mechanisms occurring in the gut and leading to the release of peripheral signals (e.g., gut hormones) implicated in the short-term regulation of food intake are still unclear. In the context of obesity and type 2 diabetes mellitus (T2DM) management, protein intake has revealed interesting positive effects on glycemia decrease, insulin secretion, and body fat loss (10). So far, the beneficial effects of protein intake on energy homeostasis remain partially elucidated but have been mainly attributed to amino acid composition (6). Bioactive peptides have emerged as potential molecules accounting for the positive effects of protein intake on weight loss and glycemia management. The process of gastrointestinal (GI) digestion is able to release bioactive peptides at circulating levels that might exert significant physiological effects on energy homeostasis. Unfortunately, their quantification *in vivo* still remains challenging. Some food protein-derived peptides, especially from dairy proteins, have demonstrated several biological activities, and these have been well characterized in relation to glycemia management (11). Nevertheless, the many bioactivities of food-derived peptides described so far still need to be better defined and integrated in a context of physiological function. Here, we review the involvement of protein-derived bioactive peptides in the short-term regulation of food intake and the mechanisms of protein-induced satiety, with a special focus on the gut hormones, cholecystokinin (CCK), and glucagon-like peptide 1 (GLP-1) on the one hand, and some aspects of glucose homeostasis on the other hand.

## CCK Secretion and Bioactive Peptides

Cholecystokinin, mainly secreted by enteroendocrine I cells located in the upper intestinal tract, acts at different levels on food intake regulation. It retards gastric emptying, stimulates pancreatic secretion and decreases food intake. Several studies in rats or humans have proved that protein or protein hydrolyzate intake could stimulate CCK secretion correlated with a gastric emptying decrease (12, 13), inhibit intraluminal protease activity (14) or decrease food intake (15). The GI digestion process appears as a key step which emphasizes the satiating properties of dietary proteins. Several *in vivo* and *in vitro* studies with intact proteins, their hydrolyzates or corresponding amino acid mixtures illustrate this phenomenon. Indeed, peptides are sequentially released throughout GI digestion and are, with fatty acids, the main stimuli of CCK release. Sharara et al. have shown that a protein intake stimulated postprandial secretion of CCK in rats, though indirectly, whereas free amino acid intake had no effect (16). Soy protein or casein intake in rats caused a delay in food intake decrease compared to the one induced by the respective protein hydrolyzates. This might be due to a slower release of peptides occurring during intact protein GI digestion (17). *In vitro*, the STC-1 murine enteroendocrine cell (EEC) line is widely used for intestinal hormones synthesis and secretion studies. Using this model, the greater CCK stimulating potential of various peptones or protein hydrolyzates than the equivalent mixtures of free amino acids has been shown and further investigated. Amino acid mixtures representing the composition of various protein hydrolyzates such as soy protein (18), blue whiting or shrimp (19, 20) or various animal peptones (21) displayed lower CCK enhancing effects than their associated hydrolyzates. A beneficial effect of a longer pepsin hydrolysis time has been observed on the CCK enhancing potential of a soy protein hydrolyzate (18). The peptide structure thus seems a key determinant in the stimulation of CCK secretion, although this is still questionable (22). This brings light to the central role played by the GI digestion process in generating bioactive peptides from ingested dietary proteins. Proteins preloads studies have proved to decrease food intake during meals and to faster induce satiety. Interestingly, a preload of whey proteins administrated to healthy subjects significantly decreased food intake and stimulated satiety compared to a preload of caseins, and this has been partially linked to a higher plasmatic CCK level (8). Thus, the type of protein source seems to influence the CCK enhancing potential, but this still needs to be clearly demonstrated.

**Figure 1:**
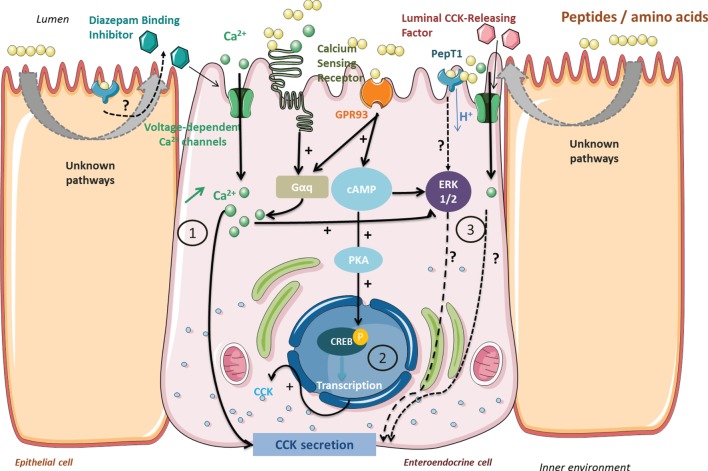
**Signaling pathways activated by peptides and amino acids involved in cholecystokinin (CCK) secretion and synthesis in enteroendocrine cells**. Peptides from protein gastrointestinal digestion released in the lumen stimulate CCK secretion *via* (1) calcium-sensing receptor (CaSR) or GPR93 activation causing an intracellular Ca^2+^ increase. Voltage-dependent Ca^2+^ channels enable an extracellular Ca^2+^ uptake when activated by CaSR and GPR93 or by membrane depolarization following dipeptide transport by PepT1. GPR93 activation by peptides may initiate CCK gene transcription (2) by ERK 1/2 or phosphokinase A signaling pathway activation. Other pathways are still investigated (3) and might indirectly imply PepT1 or luminal CCK-releasing factor in CCK secretion.

Once released into the lumen, peptides come in contact with the brush border barrier where they can stimulate gut hormone secretion. All the known different pathways have been summarized in Figure 1.

Nishi et al. have isolated a peptide fragment of soy β-conglycinin (β 51–63) able to induce food intake decrease in rats correlated to enhanced CCK levels. This fragment showed *in vivo* to have the strongest ligand affinity for a rat intestinal membrane (estimated by surface plasmon resonance) compared to other β-conglycinin fragments whose CCK enhancing potentials were lower (23). The high occurrence of arginine residues in this particular bioactive fragment could partially account for the CCK enhancing effects (13). Concomitantly, a pork hydrolyzate showed a very high ligand affinity with rat brush border membrane correlated to a dose-dependent CCK enhancing effect on the murine STC-1 cell line. Moreover, an orogastric preload of this pork hydrolyzate significantly reduced food intake in rats (24). Dietary peptides could directly stimulate CCK secretion in I cells, or indirectly in the mucosa involving intermediate factors such as luminal CCK-releasing factor (LCRF) (25). Originally purified as a 70–75 amino-acid residue peptide from rat jejunum secretion (26), LCRF was found at the highest levels in the small intestine but is present in different parts throughout the GI tract (27). LCRF was identified after several studies showing that CCK release and pancreatic secretions were inhibited by trypsin, chymotrypsin, and elastases implying an intraluminal factor, sensitive to proteases, that elicits CCK secretion (28). Early studies tested the bioactivity of different LCRF fragments and highlighted the activity of fragment 11–25 but not 1–6 for instance, in accordance with the susceptibility of LCRF bioactivity to intestinal and pancreatic enzymes degradation (29). Further, it has been shown that LCRF acts directly on CCK-secreting cells also *via* an increase in intracellular calcium at least involving the L-type calcium channel (25). The intestinal mucosa possesses a wide variety of cells in addition to the EECs, which might be stimulated by peptides and be involved in CCK secretion. Receptors and signaling pathways involved have only been partially characterized so far. Intracellular calcium mobilization has been first pointed out using *in vitro* cell lines. Némoz-Gaillard et al. have demonstrated that egg white albumin peptones stimulated CCK secretion *via* a toxin pertussis sensitive G protein inducing a Ca^2+^ cytosolic input through voltage-dependent Ca^2+^ channels in STC-1 cells (30). Activation of Ca^2+^ channels can be the first step of the signaling pathway leading to CCK secretion: L-type channels are activated by diazepam-binding inhibitor (DBI), which has been isolated from rat intestinal mucosa, inducing CCK secretion (31). GPR93, also known as GPR92, is part of the G protein-coupled receptors (GPCR) investigated for their possible link between nutrient sensing and the transduction to GI cell functions. It is highly expressed in the intestine and has been found to respond to a protein hydrolyzate in rat enterocytes and non-tumorigenous rat enterocytes cell line (hBRIE380) (32). GPR93 is also endogenously expressed by STC-1 cells where its overexpression and activation by peptones lead to increases in CCK transcription and release (33). Further investigation of the transduction pathway revealed the involvement of G_α_ proteins, a dose-dependent intracellular Ca^2+^ increase and the ERK 1/2. The calcium-sensing receptor (CaSR) is the other receptor involved in luminal peptide detection linked to CCK secretion stimulation. Part of the C family of GPCR, CaSR possesses an N-terminal Venus fly trap (VFT) domain located in the extracellular side rich in cysteine residues (34). CaSR is activated by various metabolites, extracellular Ca^2+^, and basic l-amino acids for which the VFT domain is required. CaSR is expressed in numerous tissues including the GI tract and is involved in calcium metabolism (35). CaSR is implicated in the stimulation of CCK secretion in the presence of l-phenylalanine, a well-known CCK secretion stimulator, in STC-1 cells (36). CaSR phenylalanine activation induces an intracellular Ca^2+^ mobilization ending up with CCK secretion (37). Peptide β 51–63 from β-conglycinin, a CCK-enhancing stimulator in STC-1 cells, provokes an intracellular Ca^2+^increase mediated by CaSR (38). The authors later demonstrated that CaSR was also involved in protein hydrolyzate detection and CCK secretion stimulation. Treating cells with a specific CaSR antagonist significantly affected the CCK response in the presence of protein hydrolyzates (39). Even though protein hydrolyzates contain a significant part of free amino acids, low molecular weight peptides (>1,000 Da) have been suggested to be the best stimuli of CCK secretion *via* CaSR activation. However, to the best of our knowledge, no peptide sequence has been characterized as CaSR specific.

Dietary peptides influence CCK secretion stimulation at different levels, but they also turn out to be influencing CCK gene transcription. Thus, Cordier-Bussat et al. showed that meat and egg albumin peptones had a dose-dependent effect on CCK secretion stimulation in STC-1 cells but also on the mRNA levels of the CCK gene (21). The authors later proved that peptones were able to stimulate cAMP release and to promote phosphokinase A (PKA) activation that induces CREB transcription factor phosphorylation, activating the CCK gene promoter in STC-1 cells (40). Choi et al. also noticed in STC-1 cells that GPR93 activation by peptones, or a specific agonist, led to an increase in CCK mRNA levels. Peptones were able to activate the PKA pathway that promoted the activation of the CCK gene promoter, and this has not been stated with the specific agonist. GPR93 activation by oligopeptides activates several signaling pathways that might influence both CCK synthesis and secretion (33). Indeed, Choi et al. studies in the STC-1 model implicated the ERK1/2 (MEK), PKA, and calcium/calmodulin-dependent protein kinase (CaMK) pathways in the mediation of CCK upregulation. Furthermore, Gevrey et al. work, also in STC-1 cells, had already shown peptone-induced involvement of the cAMP, PKA, and CREB as the primary pathway, together with a Ca^2+^ dependent ERK1/2 (MEK) pathway and a minor involvement of CaMK on CCK gene promoter activity. They demonstrated a total inhibition of this promoter activity when all pathways were blocked, suggesting crosstalk between them. Previous evidence of possible interactions between the cAMP and ERK pathways in different cell types also exists (41–43).

Anorexigenic effects of dietary peptides are also mediated by peripheral CCK-1R (13, 17, 44). Raybould et al. have proved that luminal nutrients stimulated CCK secretion that activates vagal afferents and inhibits gastric emptying (45). Later, Darcel et al. have pointed out that the di/tripeptides transporter PepT1 was also implied in the CCK secretion signaling pathway. The authors demonstrated that a duodenal infusion of meat peptones led to a vagal afferent discharge inhibited by a PepT1 inhibitor infused in the duodenal mucosa (46). The indirect role of PepT1 in CCK secretion induced by protein hydrolyzates was clearly pointed out in STC-1 cells as well as in native human intestinal I cells. Indeed, these cells were activated by PepT1 agonists, but this effect was not associated with CCK secretion alteration and was not affected by PepT1 antagonist treatment (47). These authors thus excluded a direct role of PepT1 in mediating the effect of peptone on CCK secretion. To account for an indirect role of PepT1, it was suggested that this transporter on enterocytes could promote a signaling factor release like the DBI that would trigger CCK release by I-cells. Moreover, although these authors found PepT1 transcripts in these cells, STC-1 expression of PepT1 could not be confirmed by another group (48). Remarkably, dietary peptides can also behave as CCK-1R agonists: soy or potato protein hydrolyzates known as CCK secretion stimuli in STC-1 cells, additionally act as partial agonists of CCK-1R in CCK-1R-overexpressing CHO cells. In the case of soy protein hydrolyzate, Staljanssens et al. demonstrated that the β-conglycinin hydrolyzates generated by GI digestion partially activate CCK-1R in CHO-CCK-1R cells but also probably other receptors involving an intracellular calcium response. Indeed, elevation of intracellular calcium was also noted in the native CHO cells, and more puzzling, this effect was decreased in the presence of a CCK-1R antagonist in both cell types (49). As the intestinal mucosa is densely innervated, vagal afferents expressing CCK-1R could be accessible to luminal content and be directly activated by dietary peptides (50). Lately, a study in vagotomized pigs has questioned the predominant role of the vagus nerve. CCK-1R blockade in abdominal vagal afferents did not abolish plasmatic CCK level increase and satiety after a liquid meal (51). This highlights that other peripheral CCK-1R could be involved and might have a greater role than the ones located in vagal afferent neurons.

To summarize, dietary peptides activate distinct signaling pathways involved in CCK secretion that promotes satiety and decrease food intake. They act in EECs by activating specific receptors (GPR93, CaSR) that, in response, induce CCK secretion stimulation *via* intracellular calcium mobilization. Peptides may indirectly act on the intestinal mucosa and stimulate the secretion of intermediate factors (LCRF) inducing CCK secretion in EECs. Another pathway stimulated by dietary peptides might involve PepT1 but has not been fully characterized yet. Peptides may also interact with CCK-1R either as partial agonist in vagal afferents located in the intestinal mucosa or indirectly by activating a PepT1 involving signaling pathway. Finally, peptides regulate CCK synthesis at the CCK gene transcription level, but the pathways involved have to be further elucidated.

## GLP-1 Secretion and Bioactive Peptides

**Figure 2:**
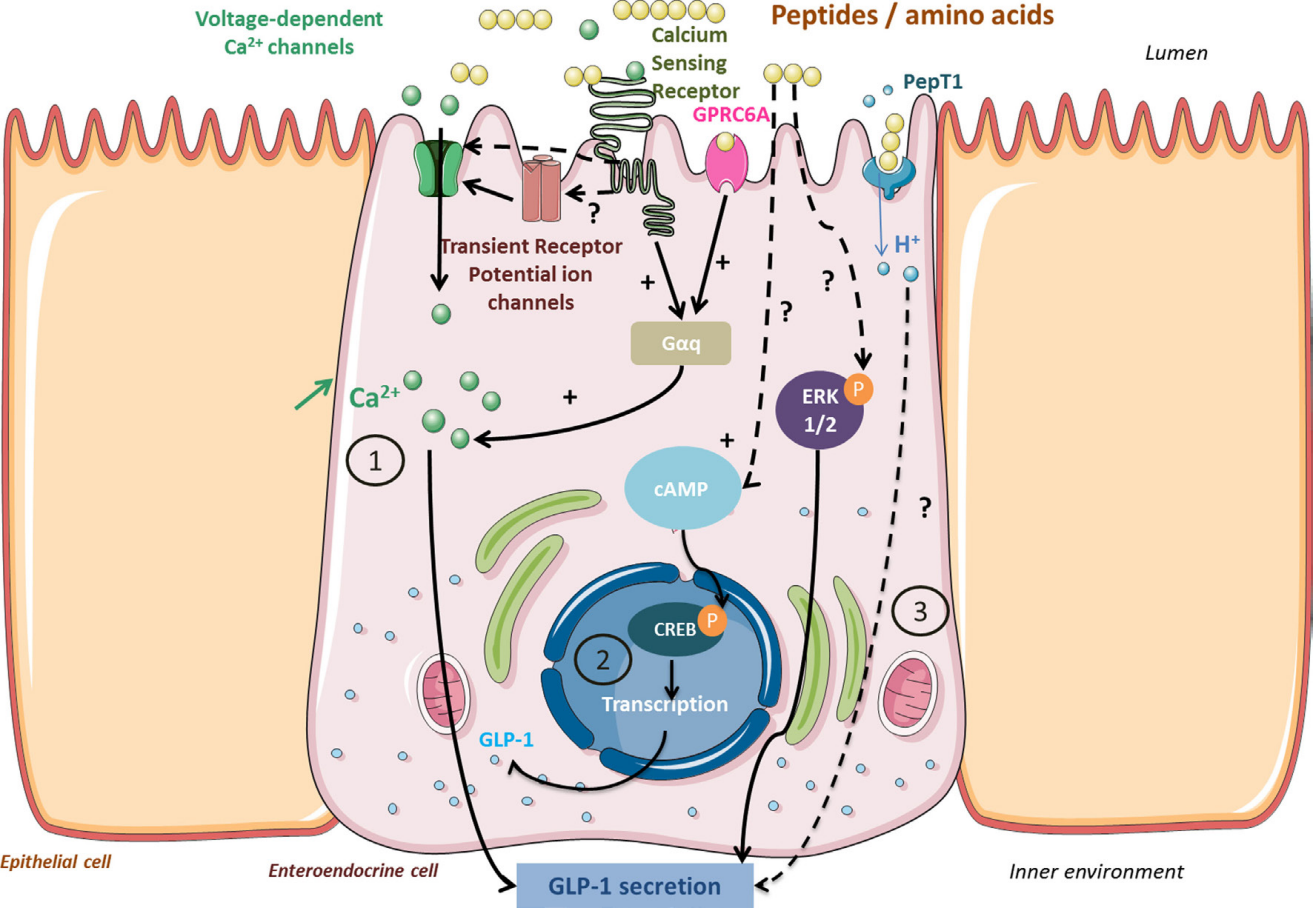
**Signaling pathways activated by peptides and amino acids involved in glucagon-like peptide 1 (GLP-1) secretion and synthesis in enteroendocrine cells**. Peptides from protein gastrointestinal digestion released in the lumen stimulate GLP-1 secretion *via* calcium-sensing receptor (CaSR) or GPRC6A activation (1). In return, they activate a Gαq subunit that activates PLC- and IP3-dependent signaling pathways and provokes an intracellular Ca^2+^ increase. Activation of Ca^2+^ channels by CaSR or transient receptor potential channels enables Ca^2+^ uptake. Peptides may also activate proglucagon gene transcription *via* a cAMP-dependant pathway leading to CREB phosphorylation (2). Unknown pathways involved in GLP-1 secretion might involve ERK 1/2 phosphorylation or proton uptake coupled to peptide transport in PepT1 (3).

Glucagon-like peptide 1 plays a significant role in energy homeostasis: it regulates blood glucose *via* its incretin action and promotes satiety and food intake decrease *via* its anorexigenic properties. That is why GLP-1 has recently emerged as an interesting therapeutic target in T2DM and obesity treatment approaches. Positive results from bariatric surgery on T2DM and obese subjects (sustainable weight loss, blood glucose regulation improvement) were partially attributed to elevated plasmatic GLP-1 levels, but these still remain partially unresolved (52). Dietary protein intake is one of the stimuli of GLP-1 secretion in EECs of the L-type, more abundant in the distal intestine, and activates several signaling pathways (Figure 2). GLP-1 effects were described after dietary protein intake from either animal sources, especially milk-derived proteins (53) or plant sources (54). A whey protein load before a meal led to a faster food intake decrease and satiety stimulation correlated to higher circulating GLP-1 levels in healthy subjects (55). A high-protein diet significantly increased postprandial GLP-1 levels compared to a conventional protein diet in healthy subjects, and extended satiety was partially attributed to these elevated GLP-1 levels (56). A preload of blue whiting administered to rats induced a short-term food intake decrease correlated to a plasmatic CCK and GLP-1 level increase (20). Beyond their satiating properties, dietary proteins can also improve blood glucose *via* GLP-1 secretion stimulation and plasmatic dipeptidyl peptidase IV (DPP-IV) activity inhibition (57–60). Whey proteins are a well-known source of bioactive peptides stimulating GLP-1 secretion, inhibiting plasma DPP-IV activity, and stimulating insulin secretion in pancreatic cells (61). However, the GLP-1-enhancing potential of proteins was found weaker than other macronutrients since lipid- or carbohydrate-based meals led to higher GLP-1 levels than after a high-protein diet (62). Moreover, an increase of the plasma GLP-1 level is not always associated with satiating effects (63). The reproducibility of GLP-1 satiating effects seems to strongly rely on several parameters such as the physiological state of the patient or experimental conditions of the study like the presence of other macronutrients, the protein source, and the delay duration after preload administration. This tends to make the comparison between different studies delicate (64). Regarding the secretion trigger mechanisms of GLP-1, two ways have been uncovered that explain the biphasic secretion of GLP-1. First, the activation of vagal afferents located in the duodenum, which indirectly stimulates GLP-1 secretion in distal EECs, then a direct contact with the EECs located in the ileum (65, 66). *In vitro* cell models have been widely developed to better understand the mechanisms of nutrient chemosensing. Animal (meat, egg white albumin) or plant protein (zein, rice) hydrolyzates have demonstrated GLP-1 enhancing properties in murine EEC lines such as STC-1 (67) and GLUTag (60, 66) or human cell lines such as NCI-H716 (68). Free amino acids also have GLP-1 enhancing properties, but the resulting effect appears lower than for peptides (20, 67). Mechanisms of GLP-1 secretion triggered by free amino acids have been deeper investigated. l-Glutamine induces membrane depolarization and activation of a metabolic pathway involving intracellular calcium mobilization in GLUTag cells (69). This pathway has later been confirmed in primary intestinal cells where l-glutamine-induced membrane depolarization was associated to cAMP and intracellular calcium increases, probably mediated by a GPCR (70). However, in both healthy and T2DM patients, encapsulated l-glutamine ingestion did not influence GLP-1 levels to significantly induce beneficial metabolic effects. Surprisingly, l-glutamine intake was even followed by food intake increase and suggested that l-glutamine might interact with orexigenic pathways (71). CaSR, preferentially activated by aromatic amino acids and expressed in EECs, is one of the receptors involved in the GLP-1 secretion pathway. Indeed, amino acids such as phenylalanine, tryptophan, glutamine, or asparagine have shown a GLP-1 enhancing effect in isolated rat intestines, and this was strongly altered by a specific CaSR antagonist (72). Another GPCR, of the class C, named GPRC6A has been characterized as an amino acid chemodetector more sensitive to basic amino acids exhibiting hydroxyl or sulfuryl groups. Extracellular binding of l-ornithine with GPRC6A triggered GLP-1 exocytosis by activating the intracellular calcium and inositol triphosphate related pathway in GLUTag cells (73).

Activation pathways triggered by peptides are under investigation but display certain similarities with those activated by amino acids such as intracellular calcium increase. One tetrapeptide of glycine residues stimulates GLP-1 secretion in NCI-H716 cells associated to intracellular calcium increase (74). Two distinct peptide sensing pathways have been highlighted in native L cells, one involving CaSR activation and intracellular calcium variation and the other peptide transport by PepT1 associated with membrane depolarization (75). Other transporters are involved in calcium regulation, such as voltage-dependent Q type channels or transient receptor potential channels and might be activated by protein hydrolyzates. Thus, they might participate in the GLP-1 secretion as suggested in a study realized in murine native EECs (76). Another intracellular signaling pathway has been characterized in NCI-H716 cells and involves MAP kinase metabolites: ERK1/2 phosphorylation activated by meat peptones triggers GLP-1 secretion (77). Finally, dietary peptides in the form of protein hydrolyzates are also able to positively influence proglucagon gene transcription in both STC-1 and GLUTag cells (67, 78) by cAMP increase and CREB transcription factor phosphorylation (79). To the best of our knowledge, the tetra-glycine peptide was the only peptide sequence known for its GLP-1enhancing properties until recently; our group identified three peptides, obtained from the GI digestion of bovine hemoglobin, able to highly stimulate GLP-1 secretion in STC-1 cells: ANVST, TKAVEH, and KAAVT (80).

## Bioactive Peptides and DPP-IV Activity: GLP-1 Activity Regulation and Indirect Effect on Glucose Homeostasis

Dipeptidyl peptidase IV is a serine exopeptidase that removes dipeptides from the N-terminal side of substrates, including GLP-1 and GIP, by cleaving post-proline or -alanine residues (81). It cleaves and *de facto* quickly inactivates GLP-1 following its secretion and therefore appears as a strong inhibitor of its activities (82). DPP-IV exists in transmembrane and soluble active forms and is expressed in various tissues and fluids. It has also been implicated in many other regulatory processes by its interaction with neuropeptides or chemokines (83). Today, DPP-IV inhibitors are thus considered an advanced class of agents for T2DM management due to their effects on the GLP-1 availability and recovery of the incretin effect. In this way, the oral administration of DPP-IV inhibitors (gliptins) is the most recent alternative treatment of T2DM (84). However, numerous works have pointed out the advantage to identify “natural” as in food-derived peptide inhibitors of DPP-IV activity as an alternative for synthetic inhibitors to reinstate the incretin effect in T2DM. GI dietary protein digestion is a natural enzymatic hydrolysis release of bioactive peptides that could exhibit DPP-IV inhibitory potentials close to those of peptides released under controlled enzymatic hydrolysis. IC_50_ values of various digests generally range from 1 to 5 mg·mL^−1^ like numerous protein hydrolyzates. As an example, several milk protein digests, generated under *in vitro* conditions, reached similar IC_50_ values compared to protein hydrolyzates obtained with microbial enzymes such as Alcalase^®^ or Flavourzyme^®^ (85). Gruyere GI digestion has shown to be an interesting source of Ile–Pro–Ala, and Val–Ala–Pro–Phe–Pro–Glu–Val, two DPP-IV inhibitory peptides (86). Alaska pollock (*Theragra chalcogramma*) skin collagen, digested under *in vitro* GI conditions, has IC_50_ values ranging from 1 to 2 mg·mL^−1^ (87), and similar values have been measured with salmon collagen hydrolyzates obtained with a controlled enzymatic hydrolysis (88). A tetrapeptide Val–Ala–Ala–Ala has been recently isolated from an *in vitro* GI digest of bovine hemoglobin with an IC_50_ of 0.141 ± 0.014 mM (80). A similar trend has been observed with plant protein GI digests. Amaranth (*Amaranthus hypochondriacus*) seed digests obtained by GI digestion have IC_50_ values close to 1 mg·mL^−1^ (89). Cowpea bean GI digestion (*Vigna unguiculata*), germinated or non-germinated, has produced digests with DPP-IV inhibitory properties at 0.58 mg·mL^−1^ soluble protein. Two peptides Thr–Thr–Ala–Gly–Leu–Leu–Gln and Lys–Val–Ser–Val–Val–Ala–Leu, characterized by LC-MS-MS in these isolated digests, could have interesting DPP-IV inhibitory properties. A docking study has revealed that these two peptides could strongly interact with the catalytic site of the DPP-IV (90). The process of GI digestion could be able to naturally generate bioactive peptides from dietary proteins with DPP-IV inhibitory properties. The inhibitory potential seems to increase along the progress of digestion: most of the intestinal digests of *in vitro* GI hemoglobin digestion exhibited lower IC_50_ values than those of gastric digests (78). However, most of the studies focus on investigating DPP-IV inhibitory properties of protein hydrolyzates during their GI digestion. When digested, protein hydrolyzates often exhibit better DPP-IV inhibitory potentials than those of native proteins. This has been noticed with cuttlefish (91, 92), rice, pea, soy, hemp protein (92), or whey protein hydrolyzates (93). A similar observation has been made when comparing the DPP-IV inhibitory potential of cow’s milk yogurt from microbial fermentation and its respective GI-derived digests. The DPP-IV inhibitory potential of this yogurt GI digests was significantly better than the yogurt one and was constantly progressing over digestion time (94). GI digestion extends protein degradation and, as a consequence, promotes the release of new potential bioactive peptides. In that sense, most of the studies first focus on optimizing hydrolysis conditions to generate bioactive peptides and then investigate peptide or hydrolyzate stability and their associated bioactivities in simulated GI conditions. Bioactive peptides may be released exogenously (enzymatic hydrolysis and fermentation) or endogenously (GI digestion of dietary or endogenous proteins), but they need to survive GI conditions and to be absorbed, implying crossing the intestinal barrier, to exert their inhibitory potentials on circulating DPP-IV that would impact the most GLP-1 activity. Indeed, GI conditions may compromise their bioavailability and bioactivity. Thus, simulating *in vitro* GI digestion is a crucial preliminary step to predict the *in vivo* stability of peptides or protein hydrolyzates in proteolytic conditions. Three peptides were isolated from macroalga hydrolyzates (*Palmaria palmata*) Ile–leu–Ala–Pro, Leu–Leu–Ala–pro, and Met–Ala–Gly–Val–Asp–His–Ile and proved to keep their DPP-IV inhibitory properties after simulating gastric and intestinal digestion conditions (95). One fraction isolated from an α-lactalbumin hydrolyzate was not affected in terms of DPP-IV inhibitory properties after simulating GI digestion (1.20 ± 0.12 mg·mL^−1^). Nevertheless, characterizing peptide sequences from various bioactive fractions (digested or not) by LC-MS-MS led to the conclusion that the DPP-IV inhibitory effect observed did not necessarily involve the same sequences before and after simulating GI digestion of the fractions (96). The action of GI enzymes can generate new sequences that might also reveal greater DPP-IV inhibitory properties than the native peptide. This was noticed for three peptides released from a cooked tuna juice hydrolyzate (*Thunnus tonggol*) obtained by enzymatic hydrolysis. Their DPP-IV inhibitory properties were enhanced after simulating GI digestion (97). Recently, a couple of studies have been investigating the potential bioactivity of endogenous peptides. Endogenous proteins represent a noticeable protein intake, and they are also degraded by GI digestion. Like dietary proteins, they can be regarded as a potential source of bioactive peptides. A human serum albumin hydrolyzate has exhibited DPP-IV inhibitory effects that remained in enriched fractions and lysozyme GI digest of the same hydrolyzate (98). Two inhibitory peptides from endogenous proteins, predicted by *in silico* digestion, have confirmed *in vitro* their potentials: Met–Ile–Met from human serum albumin (IC_50_ = 800.51 ± 4.90 µM) and Arg–Pro–Cys–Phe from endoribonuclease (IC_50_ = 1,056.78 ± 61.11 µM). Although their IC_50_ values do not indicate a strong inhibitory potential compared to Ile–Pro–Ile, endogenous proteins are a complementary source of bioactive peptides (99).

Thus, dietary protein, protein hydrolyzate, or dietary peptide intake could be part of T2DM therapeutic approaches by specifically targeting DPP-IV activity. To date, few *in vivo* studies have confirmed DPP-IV inhibitory potentials measured *in vitro*. Studies in streptozotocin-induced obese, Zucker diabetic fatty rat, or lean rats have pointed out that protein hydrolyzate or peptide intake could improve blood glucose, circulating GLP-1 and insulin levels and also decrease plasma DPP-IV activity. This has been described with various hydrolyzates from zein (58), pork gelatin skin (100), salmon gelatin (59), and tilapia gelatin (101). The peptide Leu–Pro–Gln–Asp–Ile–Pro–Pro–Leu, a β-casein fragment isolated from Gouda cheese, exhibited a high DPP-IV inhibitory potential *in vitro* (IC_50_ = 46 µM) and significantly improved blood glucose in diabetic rats after an oral glucose tolerance test. However, the authors did not specify whether this effect was related to DPP-IV activity inhibition (102). Indeed, protein and protein hydrolyzate intake may also improve blood glucose in diabetic rats without reducing plasma DPP-IV activity. In diabetic rats, plasma DPP-IV activity remained higher than in control rats after a protein rich 6-week diet made of either casein or white egg hydrolyzate, although fasting blood glucose and circulating insulin levels were significantly improved (103).

## Bioactive Peptides and Opioid Receptors: Intestinal Gluconeogenesis (IGN) and Protein-Induced Satiety

Besides their interaction with gut hormones synthesis and secretion, food-derived peptides could interact with the peripheral opioid receptors and indirectly induce gluconeogenesis that participates in the maintenance of satiety and reduction of food intake. Peripheral opioid receptors are involved in gastric emptying inhibition and food intake-induced satiety by the release of endogenous opioid peptides that act in the CNS (104). Exogenous opioid peptides produced by the GI digestion of alimentary proteins could interact with these receptors and thus intervene in food intake regulation. Casein and soy protein ingestion induces food intake decrease mediated by two distinct signaling pathways, one involving CCK-1R receptors and the other, peripheral μ-opioid receptors (MOR). GI digestion seems to be the source of the release of peptides like β-casomorphin, derived from caseins and known for its opioid activities (17, 105). The name “nutropioids” has been coined for these opioid oligopeptides originating from the diet. Besides, it is known that products of alimentary protein digestion can act as antagonists of MOR present on afferent nerve endings in the intestinal mucosa and portal vein. Detection of these oligopeptides is transmitted to the CNS and induces a decrease in food intake. This regulatory loop comes in complement to the action of the endogenous peptides released following food intake, like endorphins, and demonstrates the plurality of pathways engaged at the peripheral and central levels to promote satiety (106, 107). Mithieux et al. described a regulatory loop of food intake implicating portal vein MOR and IGN activated by alimentary protein GI digestion. This theory rests on the anorexigenic properties of glucose: the antagonistic action of oligopeptides in the portal vein MOR activates IGN *via* a gut–brain axis increasing glycemia that in turn activates hypothalamic regions involved in food intake regulation (108–113). However, only selected dipeptides have been tested so far in these studies to validate the portal vein MOR implication and no food-derived peptide motif has to date, been identified for its anorexigenic properties through this regulatory loop. In contrast, it is noteworthy that the vast majority of proteins investigated as a source of bioactive peptides, of very different animal and plant origins, have been found to produce opioid sequences when hydrolyzed/digested. These food-derived opioid peptides have not been systematically tested for their effect on opioid receptors, but agonistic activity seems to be preponderant, with only few and exclusively from milk products, opioid peptides with antagonist activity. However, it is striking again that all these food-derived opioid peptides have been shown to have a preference for MOR (114). Albeit controversial (115), particularly regarding the importance and relevance of the IGN (high-protein diet context) in comparison to hepatic gluconeogenesis production (116) and species discrepancies (117), this model of protein-induced satiety based on the portal vein MOR and IGN elegantly brings together two critical actors in the regulation of food intake, the opioid system, and glucose homeostasis. It reinforces the central role of the gut–brain axis in energy homeostasis and especially in food intake regulation and highlights the role of the process of digestion in producing food protein-derived bioactive peptides.

## Conclusion

For decades, the process of GI digestion has been studied merely for its capacity to transform food into nutriments, the source of energy for our body. It is only recently that the GI tract has been considered a dynamic interface between the luminal environment and the internal environment. Interaction between nutriments and the intestinal barrier elicit the activation of multiple signaling pathways, including some involved in energy homeostasis regulation. With the exponential increase of people affected by diseases linked to the metabolic syndrome, alimentary proteins become the subject of increasing interest since they reduce food intake, induce satiety and increase energy expenditure. Yet, the underlying mechanisms are still not completely elucidated. The *in vitro* study of some mechanisms, notably the production and secretion of the GI hormones, highlighted the primary role of bioactive peptides originating from protein GI digestion. Regarding the existing links between these peptides and the regulation of intestinal hormones, some signaling pathways have been unveiled implicating a role for the GPCR family of receptors. Thus, the presence of these receptors on the apical side of the EECs constitutes the first level of integration of the information on the luminal content. These receptors act as chemodetectors and initiate the translation of the detected information into a hormonal response. Hence, GPCRs attract particular attention as novel targets for obesity and type 2 diabetes treatments. With regards to the peptides, very few structural criteria are known to date to favor these receptors activation.

It is nowadays admitted that the GI tract has the capacity to release bioactive peptides that participate in the regulation of energy homeostasis, from ingested alimentary proteins. While the effects of these peptides confirming a decrease in food intake and an increase in satiety have been demonstrated *in vivo*, the correlation with an increase in intestinal hormone release or DPP-IV inhibition has not often been established. The presence of the peptides in the intestinal lumen and their potential crossing of the intestinal barrier could be the trigger of other food intake decreasing signaling pathways activation, like the indirect activation of IGN by the portal vein MOR antagonism, or the stimulation of not yet studied intestinal hormones release. Finally, *in vivo* identification of the peptides produced during GI digestion and responsible for the described effects is still difficult to realize. Therefore, analytical strategies have been implemented *in vitro* in order to follow the release of peptides during GI digestion and meanwhile to reveal their bioactive potential.

## Author Contributions

JC and DD participated in all steps of preparation of this manuscript and contributed equally to this work. RR and PD participated in the editing of the manuscript and revised it critically. BC was awarded the funding and participated in the conception and in the editing of the manuscript and revised it critically.

## Conflict of Interest Statement

The authors declare that the research was conducted in the absence of any commercial or financial relationships that could be construed as a potential conflict of interest.
